# *Transthyretin* Is a Key Regulator of Myoblast Differentiation

**DOI:** 10.1371/journal.pone.0063627

**Published:** 2013-05-22

**Authors:** Eun Ju Lee, Abdul R. Bhat, Majid Rasool Kamli, Smritee Pokharel, Tahoon Chun, Yong-Ho Lee, Sang-Seop Nahm, Joo Hyun Nam, Seong Koo Hong, Bohsuk Yang, Ki Young Chung, Sang Hoon Kim, Inho Choi

**Affiliations:** 1 School of Biotechnology, Yeungnam University, Gyeongsan, Republic of Korea; 2 College of Life Sciences and Biotechnology, Korea University, Seoul, Republic of Korea; 3 Department of Biomedical Science, Catholic University of Daegu, Gyeongsan, Republic of Korea; 4 College of Veterinary Medicine, Konkuk University, Seoul, Republic of Korea; 5 Department of Physiology, Dongguk University, College of Medicine, Gyeongju, Republic of Korea; 6 Hanwoo Experiment Station, National Institute of Animal Science, RDA, Pyongchang, Republic of Korea; 7 Department of Biology, Kyung Hee University, Seoul, Republic of Korea; University of Minnesota Medical School, United States of America

## Abstract

*Transthyretin* (*TTR*) is a known carrier protein for thyroxine (T_4_) and retinol-binding protein in the blood that is primarily synthesized in the liver and choroid plexus of the brain. Herein, we report that the *TTR* gene is expressed in skeletal muscle tissue and up-regulated during myotube formation in C2C12 cells. *TTR* silencing (TTR_kd_) significantly reduced myogenin expression and myotube formation, whereas myogenin silencing (MYOG_kd_) did not have any effect on *TTR* gene expression. Both TTR_kd_ and MYOG_kd_ led to a decrease in calcium channel related genes including *Cav1.1*, *STIM1* and *Orai1*. A significant decrease in intracellular T_4_ uptake during myogenesis was observed in *TTR_kd_* cells. Taken together, the results of this study suggest that *TTR* initiates myoblast differentiation via affecting expression of the genes involved during early stage of myogenesis and the genes related to calcium channel.

## Introduction

Myogenesis is the formation of multinucleated myofiber with a contractile capacity from muscle satellite cells (MSCs). Myogenesis involves cell cycle arrest, myogenic activation, cell alignment, multiple rounds of cell fusion and an increase in size with peripheral localization of the nuclei [1]. This process is highly regulated and involves growth factors, cytoskeletal proteins, and muscle specific transcription factors such as *myogenin* (*MYOG*) and *myocyte enhancer factor-2* (*MEF2*) which are regulated by an increase in cytosolic Ca^2+^ concentration [2], [3],[4], [5], [6].

Increased intracellular calcium activates intracellular protease, calpains, calcineurine and serine-threonine phosphatase, which plays a critical role in cell migration and fusion to myotube formation [7], [8], [9], [10]. Kubo Y. [11] proposed that T-type voltage-gated calcium channel (VGCC) and inward rectifier K^+^ current increase the basal intracellular Ca^2+^ level, which may be essential to the initial stages of mesodermal stem cell differentiation. Ca^2+^ through T-type VGCC also contributes to other differentiation processes including neural differentiation [12] and neuroendocrine differentiation of prostate cancer cells [13]. Moreover, there is a great deal of evidence that Ca^2+^ influx through T-type VGCCs results in signalling that affects the expression of genes involved in cell proliferation, programmed cell death, and neuronal differentiation [14], [15], [16], [17], [18], [19], [20]. Similarly, L-type VGCC like *Cav1.1* is also present in skeletal muscle [21]. In addition to VGCC, human myoblasts can generate Ca^2+^ signals by Ca^2+^ release from inositol 1,4,5-triphosphate-sensitive Ca^2+^ stores followed by entry through store operated calcium (SOCE) channels [3]. *STIM1*and *Orai1* are essential component of store-operated Ca^2+^ entry (SOCE) that is evoked in response to a fall in Ca^2+^ in the endoplasmic reticulum. *STIM1*is a calcium sensor in endoplasmic reticulum and *Orai1* in the plasma membrane [22], [23].

*Transthyretin* (*TTR*), which exists in tetrameric form is a carrier protein for thyroxine (T_4_) and retinol-binding protein in the blood [24], [25], [26]. The liver and choroid plexus of the brain are major organs responsible for the synthesis of *TTR* in plasma and cerebrospinal fluid, respectively. In addition to the liver and brain, mRNA expression of *TTR* has been reported in the skeletal muscle of rats [27]. *TTR* gene knock-out mice increased neuropeptide Y, suggesting that *TTR* is critical in nervous system [28]. RNA interference targeting *TTR* in mammalian cells has been found to increase the initial efficacy of neural prosthetic devices before insertion [29]. We recently reported that *TTR* is induced in bovine primary MSC differentiation [30]. Herein, we investigated the role of *TTR* during myogenesis in C2C12. Silencing of *TTR* demonstrated the inability of cell alignment before fusion, leading to the formation of impaired myotubes.

## Materials and Methods

### Mouse Tissues

In this study, 6 or 18 weeks old male C57BL/6 mice were used for RNA isolation. Briefly, four week old mice were obtained from Daehan Biolink (Eumseong, Korea) and housed four per cage in a temperature-controlled room with a 12 hr light/12 hr darkness cycle. Throughout the study period, animals were allowed free access to standard rodent chow containing 4.0% (wt/wt) total fat (Rodent NIH-31 Open Formula Auto, Zeigler Bros., Inc., Gardners, PA, USA) and water. At 6 and 18 weeks of age, mice were anesthetized with sodium pentobarbital and exsanguinated. Tissue samples were then collected, quickly frozen in liquid nitrogen, and stored at −80°C until processed for RNA extraction. For immunohistochemistry, mice were anesthetized by intraperitoneal injection of tribromoethanol (Avertin, 250 mg/kg, Sigma Aldrich CA, USA) for transcardial perfusion with PBS (phosphate buffered saline) to remove the blood. The animals were then perfusion fixed with 10% neutral buffered formalin, after which solid organs and skeletal muscles from the trunk and extremities were removed and post-fixed in the same fixative overnight at 4°C. The fixed organs were then processed for routine paraffin embedding, and the paraffin-embedded tissue blocks were cut to 6-µm thick sections for immunohistochemistry. The experimental protocols for the care and use of laboratory animals were approved by the Institutional Animal Care and Use Committee of Konkuk University.

### Cell Culture

C2C12 cells, a murine myoblast cell line, were cultured in DMEM (Dulbecco’s modified Eagle’s medium; HyClone Laboratories, Logan, UT) supplemented with 10% FBS (fetal bovine serum, HyClone Laboratories) and 1% penicillin/streptomycin (Invitrogen, Carlsbad, CA, USA) at 37°C with 5% CO_2_. For differentiation, cells grown to 70% confluence were switched to differentiation media (DMEM with 2% FBS) and then cultured for 0, 2, 4, and 6 days, during which time the medium was changed every two days. Cells were treated with T4 (50 ng/ml) for 4 and 6 days. C2C12 cells were kindly provided by Korean Cell Line Bank, Republic of Korea.

### *TTR* and *MYOG* Knock-down

C2C12 cells grown in 6-well plates to 30% confluence were transfected with 1 ng of vector, *TTR* and *MYOG* shRNA construct per well using transfection reagent and transfection medium (Santa Cruz Biotechnology, CA, USA). After 3 days, the cells were treated with 2 µg/mL Puromyocin (Santa Cruz Biotechnology) for selection. Selected cells were grown upto 70% confluence before switching to differentiation media. Sequences of shRNA constructs are provided in Table S1.

### Fusion Index

Fusion index was analyzed as previously described [31], [32]. Cell nuclei were stained with Giemsa G250 (Sigma Aldrich) and pictures were captured randomly at three different spots. Further, the number of nuclei in myotubes and the total number of nuclei in cell were counted in each field. Fusion index was calculated as the percentage of total nuclei incorporated in myotubes vs. total number of nuclei.

### RNA Extraction and Real Time RT-PCR Analysis

Total RNA was extracted from the cells and tissues using Trizol™ reagent (Invitrogen) according to the manufacturer’s protocol and then stored in diethylpyrocarbonate-treated H_2_O at −80°C until used. One microgram of RNA in a reaction mixture with a total volume of 20 µl was primed with oligo (dT)_20_ primers (Bioneer, Daejeon, Korea) and then reverse transcribed at 42°C for 50 min and 72°C for 15 min. Subsequently, 2 µl of cDNA product and 10 pmoles of each gene-specific primer were used to perform PCR, which was conducted using a 7500 real-time PCR system (Applied Biosystems, Foster City, CA, USA). Power SYBR® Green PCR Master Mix (Applied Biosystems) was used as the fluorescence source. Primers were designed with the Primer 3 software (http://frodo.wi.mit.edu) using the sequence information listed at the National Center for Biotechnology Information. The detailed information about primer sequences are provided as Table S2.

### Immunocytochemistry

C2C12 cells grown in a covered glass-bottom dish were treated with differentiation medium and stained for *TTR*, *MYOG, Cav1.1*, and *Cav3.1* protein following 0, 2, 4 and 6 days of incubation. Briefly, cells were rinsed with PBS and fixed with 4% formaldehyde, after which they were permeabilized with 0.2% Triton X-100 (Sigma Aldrich). The cells were then incubated with primary antibody (rabbit polyclonal IgG *TTR* (1∶50), mouse monoclonal IgG *MYOG* (1∶50), mouse monoclonal IgG *Cav1.1* (1∶50), and rabbit polyclonal IgG *Cav3.1* (1∶50), Santa Cruz Biotechnology) at 4°C in a humid environment overnight. Secondary antibody (1∶100; Alexa Fluor 488 goat anti-rabbit and goat anti-mouse SFX kit; Molecular Probes, Eugene, OR, USA) was then applied for 1 hr at room temperature. The samples were rinsed with PBS, after which the nuclei were counterstained with 4′ 6′-diamino-2-phenylindole (DAPI; Sigma-Aldrich). Pictures were taken using a fluorescent microscope equipped with a digital camera (Nikon). Detail information about clone names of monoclonal antibodies are provided as Table S1.

### Western Blot Analysis

Cells treated with differentiation media and cultured for different time periods were subjected to Western blot analysis. Briefly, after incubation, cells were washed with ice-cold PBS and lysed in RIPA lysis buffer containing protease inhibitor cocktail (Thermo Scientific, NH, USA). Total protein was isolated by centrifugation of the lysate at 13000 rpm for 10 min at 4°C, after which the protein concentrations were determined by the Bradford method using protein assay dye solution [33]. Total protein (40 µg) heated at 90°C for 3 min with ß-mercaptoethanol (Sigma-Aldrich) was electrophoresed in 10% SDS-polyacrylamide gel and then transferred to PVDF membrane (Millipore, MA, USA). The blots were subsequently blocked with 5% skim milk in TBST for an hour and then incubated overnight with *TTR* (1∶500), *MYOG* (1∶1000) or *ß-actin* antibody (1∶2000) (Santa Cruz Biotechnology) diluted with 3% skim milk in TBS at 4°C. Blots were next washed in TBST and incubated with horseradish peroxidase conjugated secondary antibody for an hour at room temperature, after which they were washed as described above and developed using Super Signal West Pico Chemiluminescent Substrate (Thermo Scientific).

### Patch-clamp Analysis

Cells were transferred into a bath mounted on the stage of an inverted fluorescence microscope (Ti-U; Nikon Instruments, Inc., Melville, NY, USA). The bath (approximately 0.20 mL) was superfused at 5 mL/min, and voltage clamp experiments were performed at room temperature (22°C–25°C). Patch pipettes with a free-tip resistance of approximately 2.5 MOhm were connected to the head stage of a patch-clamp amplifier (Axopatch 200B; Molecular Devices, Inc., Sunnyvale, CA, USA). The pCLAMP software v.10.2 and Digidata 1440 (Molecular Devices, Inc.) were used to acquire data and apply command pulses. Whole-cell currents were recorded at 10 kHz and low-pass filtered at 5 kHz. Current traces were stored and analyzed using Clampfit v.10.2 and Origin v. 8.0 (Microcal Inc, Northampton, MA, USA). For comparison of whole-cell currents between cells, the current amplitudes were normalized to the membrane area measured by electrical capacitance. The maximum absolute value of the current obtained (in pA) was divided by the cell capacitance (in pF). The pipette solution for the whole-cell patch clamp contained 100 mM Cs-aspartate, 32 mM CsCl, 10 mM ethylene glycol-bis (2-aminoethylether)-*N,N,N′,N′*-tetraacetic acid (EGTA), 5 mM Mg-ATP, and 10 mM HEPES at pH 7.2 (titrated with CsOH). The bath solution for whole-cell recording of the VGCC contained 120 mM NaCl, 5 mM CsCl, 10 mM TEA-Cl, 10 mM BaCl_2_, 10 mM glucose, 0.5 mM MgCl_2_, and 10 mM HEPES at pH 7.4 (titrated with NaOH).

### ELISA

Cell lysates extracted from cells differentiated for different lengths of time were used for ELISA to measure the T_4_ concentration (DRG International, Inc. Margurg, Germany). Cell lysate and enzyme conjugate were added in a specific antibody-coated microtiter and then incubated for 1 hr at room temperature. The mixture was subsequently removed and washed to remove the unbound samples. After addition of the substrate solution for 20 min, the reaction was terminated by adding stop solution and the color intensity was measured using a spectrophotometer at 450 nm (Tecan Group Ltd. Switzerland).

### Immunohistochemistry

*TTR* expression in mouse tissues was evaluated by immunohistochemistry using a *TTR* antibody (Santa Cruz Biotechnology). Briefly, paraffin-embedded tissue sections were deparaffinized, hydrated, and then quenched for endogenous peroxidase activity. The sections were blocked with 5% goat serum in PBS, after which they were incubated with the *TTR* antibody (2 µg/mL, Santa Cruz Biotechnology) overnight at 4°C. The sections were incubated with biotinylated anti-goat IgG (Vector, CA, USA) and subsequently with horseradish peroxidase-conjugated streptavidin (Vector). Positive signals were visualized by adding diaminobenzidine and hydrogen peroxide as substrates. A negative control experiment was also carried out by omitting the primary antibody. Stained sections were counterstained with methyl green and then dehydrated, mounted, and examined using a light microscope.

### Statistical Analysis

The normalized expression means were compared using Tukey’s Studentized Range (HSD) to identify significant differences in gene expression. A nominal *p*-value of less than 0.05 was considered to be statistically significant. Real time RT-PCR data were analyzed by one-way ANOVA using PROC GLM in SAS package ver. 9.0 (SAS Institute, Cary, NC, USA).

## Results

### *TTR* Expression and Localization in Mouse Tissues

**Figure 1:**
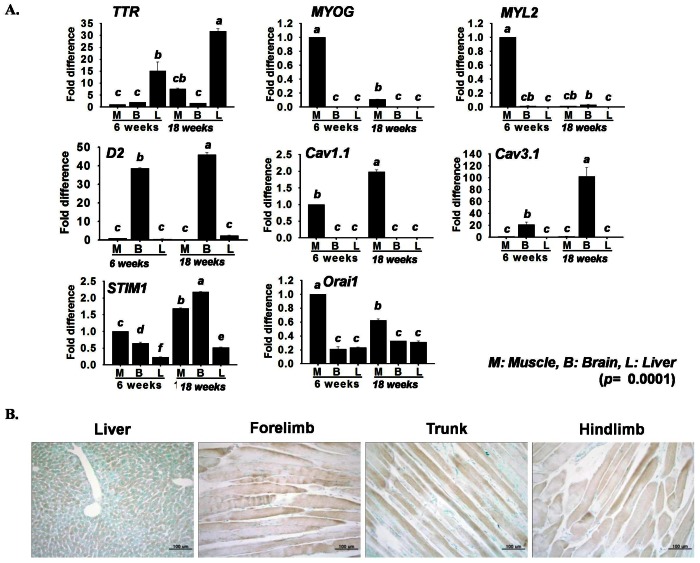
*TTR* gene expression in different mouse tissues. A) mRNA expression showed in three different tissues, M (muscle), B (brain) and L (liver), by real time RT-PCR. *TTR, STIM1* and *Orai1* were found to be expressed in all tissues, while expression of *MYOG, MYL2, D2* (*Deiodinase 2*), *Cav1.1* and *Cav3.1* genes was specific to individual organs. B) *TTR* protein detection by immunohistochemistry revealed its presence in skeletal muscles of the forelimb, hind limb and trunk, as well as in the liver. The liver was used as a positive control (n = 3). The *p* value indicates the statistical significance of the data and different letters indicate significant difference among groups.

*TTR* mRNA expression was observed in liver, muscle and brain tissues of different aged mice. The mRNA expression of the *TTR* gene was higher in 18 week old mice than 6 week old mice in muscle and liver tissues, with higher values being observed in liver tissue. The myogenesis marker genes, *MYOG* and *MYL2* (*Myosin light chain 2*), being marker genes were expressed in higher levels and at earlier stages in muscle tissues. Moreover, *Cav1.1*, *STIM1* (*Stromal interaction molecule 1*) and *Orai* (*Calcium release-activated calcium channel protein 1*) were found to be highly expressed in muscle tissues (Fig. 1A). All the skeletal muscles stained for *TTR*, including forelimb, hind limb and trunk muscles, showed higher levels of *TTR* expression (Fig. 1B). Although the *TTR* expression was observed within the cytoplasm, but the intensity was not even throughout the muscle fibers.

### *TTR* is Expressed during Myoblast Differentiation

**Figure 2:**
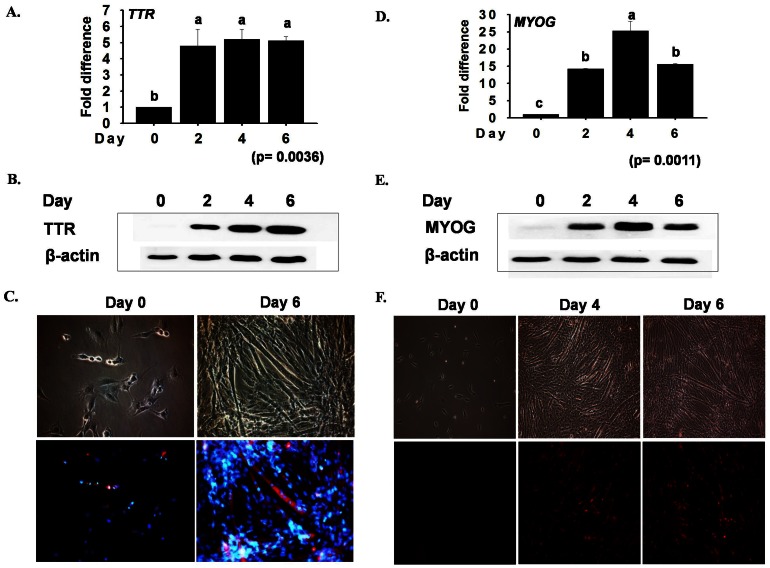
*TTR* and *MYOG* in C2C12 differentiation. C2C12 cells grown to 70% confluence and serum starved for 2, 4 and 6 days were used for mRNA and protein expression analysis. A) *TTR* mRNA showed up to a 5 fold difference in expression at day 2–6 upon real time RT-PCR analysis. B) Immunoblot analysis of *TTR* and ß-actin expression revealed low initial protein expression followed by a gradual increase with time. C) Distribution of *TTR* expression in cell cytoplasm as observed by immunostaining. The expression level was significantly upregulated at day 6. D) Time course study of mRNA expression of *MYOG* by real time RT-PCR. The maximum expression level was observed on day 4 of differentiation. E) Total proteins extracted from cells at different time points revealed very low expression of *MYOG* at the basal level that gradually increased up to day 4, then decreased at day 6. F) Cells differentiated and stained against the antibody of *MYOG* displayed the highest nuclear staining on day 4. The *p* value indicates the statistical significance of the data and different letters indicate significant difference among groups.

To investigate the role of *TTR* during C2C12 myoblast differentiation into myotubes, cells were cultured and differentiated at different time points, after which their mRNA expression was analyzed by real time RT-PCR. The data after normalization revealed a more than 4 fold increase in the expression level of *TTR* at day 2, which remained almost constant to day 6 (Fig. 2A). The expression level of proteins was verified by Western blot and immunostaining (Fig. 2B, 2C). The cells were confirmed to be in the stage of myotube formation by checking the mRNA and protein expression of *MYOG* at different time points during cell differentiation (Fig. 2D, 2E, 2F).

### *TTR* Knock-down Affects Myotube Formation

**Figure 3:**
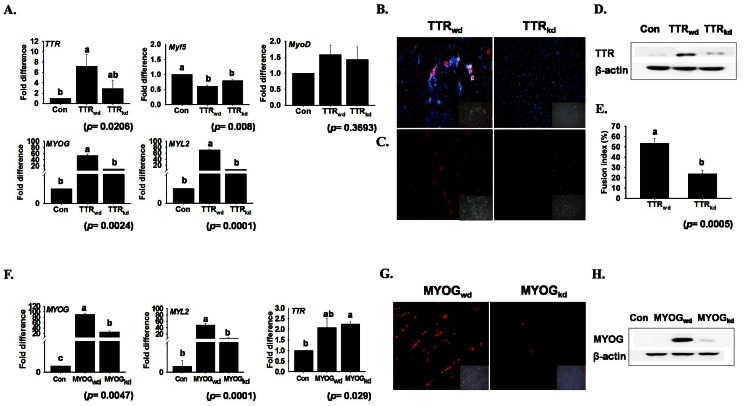
*TTR* and *MYOG* knockdown and their effect. TTR knock-down cells (TTR_kd_) showed altered mRNA expression of myogenic genes. A) A decrease in mRNA expression was observed on day 6 of transfection in *TTR* and day 5 in *MYOG* and *MYL2.* However *Myf5* and *MyoD* showed little enhancement. Control indicates the time at which cells were transfected (n = 3). B) Immunostaining of cells transfected with either TTR_wd_ or TTR_kd_ (day 6). A significant decrease of cytoplasmic *TTR* protein was observed in TTR_kd_ cells when compared with TTR_wd_ cells. C) Similarly, TTR_kd_ led to decreased nuclear myogenin protein expression. D) Western blot of TTR_kd_ agrees with the immunostaining results. E) Fusion index was performed with TTR_wd_ and TTR_kd_ at day 4. A significant decrease of nuclei fusion in TTR_kd_ was analyzed as compared to TTR_wd_ cells. F) mRNA expression of *MYOG* knock-down in C2C12 and its effect on *MYL2* and *TTR*, which peaked on day 4 of differentiation. *MYL2* is affected by *MYOG* knock-down, while *TTR* showed no change. G &H) immunostaining and immunoblot analysis verifying myogenin knockdown up to the protein level during differentiation at day 4. The *p* value indicates the statistical significance of the data and different letters indicate significant difference among groups.

To assess the previously unrecognized role of *TTR* during myogenesis, cells were transfected either with 1 ng of *TTR* shRNA (TTR_kd_) or vector GFP *(*TTR_wd_). As shown in Fig. 3A, TTR_kd_ significantly reduced mRNA expression of the genes involved in myogenesis at terminal differentiating stages, such as *MYOG* and *MYL2*. *Myf5* and *MyoD*, which are known to be involved in earlier stages of myogenesis, were unaffected at day 4. However, expression of *MyoD* was reduced significantly upto 75% when checked at day 2 (Fig. S1). shRNA transfection against *TTR* prevented the formation of myotubes as well as the decrease in the cytoplasmic distribution pattern of *TTR* and nuclear expression of *MYOG* protein (Fig. 3B, 3C: inset). The expression pattern obtained using immunoblot showed decreased levels of protein in TTR_kd_ cells (Fig. 3D). Fusion index calculated at day 4 after TTR_kd_ also agrees with the above observation, showing approximately 50% reduction as compared to TTR_wd_ (Fig. 3E). In contrast, *MYOG* knock-down during differentiation did not have any effect on *TTR* mRNA expression (Fig. 3F). *MYOG* knock-down was also verified at the protein level by immunostaning and immunoblot analysis (Fig. 3G, 3H).

### Voltage-gated Calcium Currents and Calcium-dependent Gene Expression

**Figure 4:**
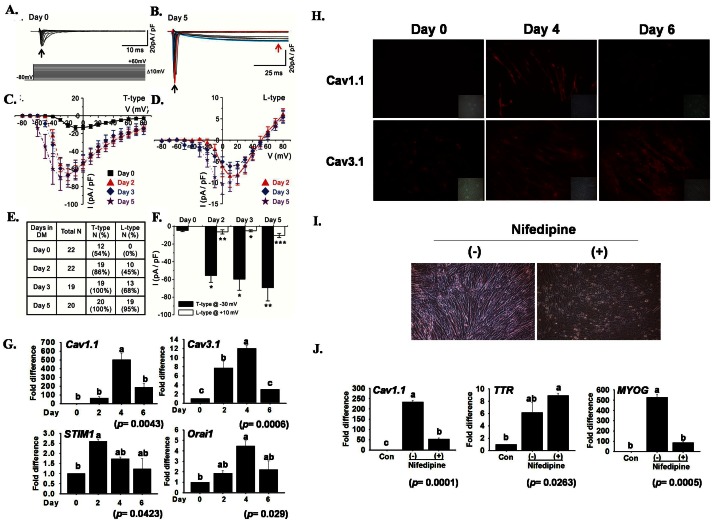
L-type and T-type calcium current and expression of Ca^2+^ channel related genes during myogenesis. Whole-cell patch clamp recordings in response to 500 ms depolarization from a holding potential (HP) of −80 mV stepped to values between −80 mV and 90 mV for 500 ms. Ba^2+^ (10 mM) was used as a charge carrier. A) Superimposition of different traces under the control conditions in response to test pulses from −80 to 40 mV. B) Representative superimposed L/T-type Ca^2+^ current traces after 5 days of culture in differentiation medium (DM). C) Average current − voltage (I–V) relationship for the initial peak Ca^2+^ current (T-type, black arrow) at 0 (n = 12), 2 (n = 19), 3 (n = 19), and 5 (n = 20) days after culture in DM. D) Average I–V relationship for the secondary peak Ca^2+^ current (L-type, red arrow) at 2 (n = 10), 3 (n = 13), and 5 (n = 19) days after culture in DM. E) Summary of the detection probabilities of L/T-type Ca^2+^ current from the patch clamp recording after culture in DM. Note that both the T- and L-type detection rate gradually increases through the time course. F) Histograms of the current density of the T-type calcium current (measured using a test pulse at −30 mV, HP −80 mV) and the L-type (measured at 0 mV). mRNA expression of Ca^2+^ channel related genes was analyzed by real-time RT-PCR. G) Up-regulated mRNA expression of calcium channel related genes during myogenesis at different time points. *Cav1.1* (L-type), *Cav3.1* (T-type) and *Orai1* showed maximum expression on day 4, whereas *STIM1* on day 2 represents the variation in calcium homeostasis at given time intervals. H) Immunofluorescence labeling indicates the distribution of *Cav1.1* and *Cav3.1* protein in the cytoplasm on days 0, 4 and 6. The highest expression of *Cav1.1* and *Cav3.1* protein was observed on day 4 and 6, respectively. I) Cells were treated with nifedipine as a L-type Ca^2+^ channel blocker (100 µM) during differentiation A change in morphology and decrease in myotube formation was observed. J) Effect of nifedipine on mRNA expression of *TTR*, *Cav1.1* and *MYOG.* Nifedipine reduced *Cav1.1* and *MYOG* expression, whereas *TTR* was unaffected (mean ± S.D., n = 3). The *p* value indicates the statistical significance of the data and different letters indicate significant difference among groups.

Calcium plays a critical role in multiple steps associated with myotube formation. In this study, to identify the functional expression of voltage-dependent Ca^2+^ channels (VDCCs) during myogenesis, the whole-cell patch-clamp recording of C2C12 cells at different time intervals was performed. Small T-type Ca^2+^ currents were detected in control cells at day 0 (Fig. 4A). However, only ∼54% of the cells expressed measurable T-type Ca^2+^ currents. After five days of differentiation, in addition to T-type current, a distinct L-type Ca^2+^ current was detected (Fig. 4B) in response to 500 ms depolarizing pulses from a holding potential of −80 mV. Fig. 4B shows representative currents recorded at −30 mV (red line), where the T-type current shows the maximum curve (black arrow), and at 10 mV (blue line) where the L-type current shows a peak current amplitude (red arrow). Fig. 4C and 4D are the individual graphs obtained from the superimposed L/T type calcium currents data (Fig. 4B). Fig. 4C shows average current − voltage (I–V) relationship for the initial peak Ca^2+^ current in T-type channel (black arrow) at different day of culture. Fig. 4D shows an average I–V relationship for the secondary peak Ca^2+^ current in L-type channel (red arrow) at different day. Mixed T- and L-type currents were recorded over the −30 mV depolarizing pulse from a holding potential. After day 2, the T-type Ca^2+^ current was detected in most of the control cells (∼86%), whereas the L-type Ca^2+^ current was detected in ∼45% of C2C12 cells. The maximum detection rate (100%) of the T-type Ca^2+^ current was attained at day 3 (Fig. 4E). The detection probability of the L-type Ca^2+^ current gradually increased during myogenesis, reaching its maximum at day 5 (Fig. 4 E). Current density through T- and L-type Ca^2+^ channels increased significantly during myogenesis (Fig. 4F, P<0.05). Although the detection probability of the T- and L-type Ca^2+^ current gradually increased during myogenesis, the T- and L-type Ca^2+^ current densities did not show a gradual increase (Fig. 4C, 4D). The size of the myotubes was significantly higher in older cultures; however, the current densities of each type of channel showed a transient increase at a specific point and increased only slightly during myogenesis. These electrophysiological data indicate that both T- and L-type Ca^2+^ channels are functionally expressed in differentiating C2C12 cells.

*Cav1.1*, *Cav3.1* and *Orai1* mRNA showed a gradual increase in expression up to day 4 (Fig. 4G). In contrast, *STIM1* peaked at day 2. Localization of protein by immunostaining revealed a similar expression pattern (Fig. 4H). Moreover, the L-type calcium channel was blocked with nifedipine (100 µM) for 4 days during myogenesis and considerable reduction in myotube formation was observed (Fig. 4I). *Cav1.1* and *MYOG* mRNAs expression were significantly reduced by blocker, while *TTR* was unaffected (Fig. 4J).

### *TTR* Controls the Calcium Channel

**Figure 5:**
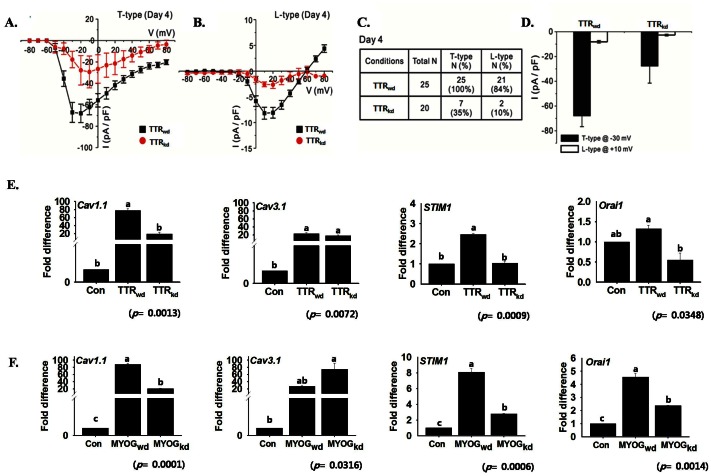
Effects of *TTR* silencing on voltage operated Ca^2+^ channels during the myogenesis. Currents were evoked by 500-ms step pulses in 10 mV increments applied from −80 mV HP (see voltage protocol in Figure 3). Current traces were obtained for every 10 mV step between −80 and 80 mV. A) I–V relationships for T-type Ca^2+^ current from TTR_wd_ (n = 25) and TTR_kd_ (n = 21). The magnitude of each current was measured on day 4 after culture in DM. B) I–V relationships for L-type Ca^2+^ current from TTR_wd_ (n = 7) and TTR_kd_ (n = 2). C) Summary of the detection probabilities of L/T-type Ca^2+^ current between TTR_wd_ and TTR_kd_ at day 4 after culture in DM. Note that the detection probability was significantly decreased in TTR_kd_. D) Histograms of the current density of the T-type calcium current (measured using a test pulse at −30 mV, HP −80 mV) and the L-type (measured at 10 mV). E & F) mRNA expression assessed by real-time RT-PCR on *Cav1.1, Cav3.1, STIM1* and *Orai1* in TTR_wd_, TTR_kd_ or MYOG_kd_ cells on day 4 of transfection. TTR knock-down resulted in a decreased effect on all calcium channel related genes. *MYOG* knock-down showed decreased expression of *Cav1.1, STIM1* and *Orai1*. Control indicates day 0 (mean ± S.D., n = 3). *p* value indicates the statistical significance of the data and different letters indicate significant difference among groups.

It was essential to identify the elements responsible for increased expression rates of T- and L-type Ca^2+^ channels during myogenesis and confirm if there was any relationship between the *TTR* and VGCCs. No specific pharmacological blocker of *TTR* was available at the time of the study; therefore, *TTR*-specific shRNA was transfected into C2C12 cells. The prevention of the regulation of these Ca^2+^ channels was validated by the whole-cell patch-clamp recordings (Fig. 5A, 5B). As shown in Fig. 5C, the detection probability of T- and L-type Ca^2+^ channels was significantly reduced by TTR_kd_ at four days after switching to the differentiation medium. The current density between TTR_wd_ and TTR_kd_ cells was compared among cells expressing T- and L-type Ca^2+^ channels. In the case of TTR_kd_ cells, a significant decrease in detection probability and the current density was observed (Fig. 5D).

The mRNA expression of T- and L-type Ca^2+^ channel subunits (Fig. 5E) along with the *STIM1* and *Orai1* was found to be highly influenced by *TTR* silencing. The reduction in expression of *Cav1.1* in TTR_kd_ cells, demonstrating its direct involvement in myogenesis. In addition, *MYOG* silencing was also found to affect Ca^2+^ entry related genes such as *STIM1, Orai1 and Cav1.1* (Fig. 5F), indicating that *MYOG* regulates these genes.

### Cell Differentiation is also Affected by T_4_

**Figure 6:**
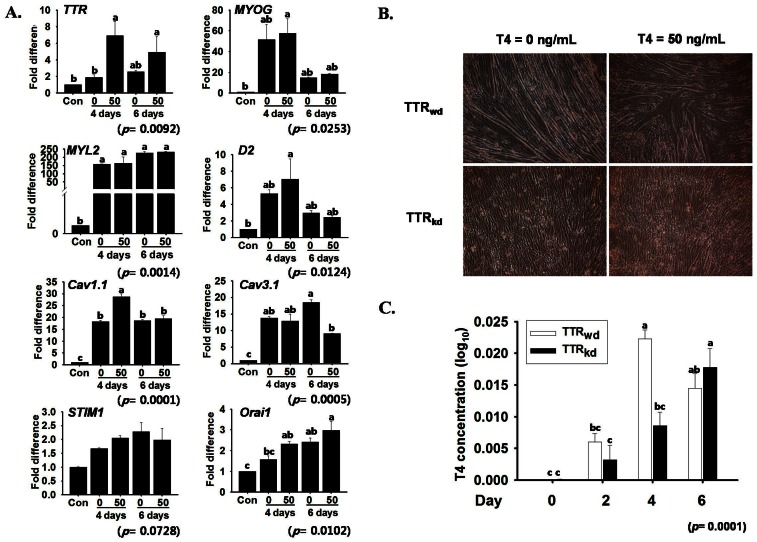
Thyroid hormone effect on myogenesis. A. mRNA expression of the *TTR* gene and genes associated with the calcium channel and myogenesis was assessed by real-time RT-PCR at day 4 and 6 of differentiation with 50 ng/mL T_4_. *TTR* expression was greatly influenced by T_4_ treatment; however, the expression of other genes related to myogenesis and the Ca^2+^ channel were variable. B) The T_4_ effect on *TTR* silencing showed no change on day 6. C) T_4_ concentration measured by ELISA in the extracts of TTR_wd_ and TTR_kd_ cells, respectively, at different days of differentiation. Control indicates the time at which proliferative media was replaced with differentiation medium. *p* value indicates the statistical significance of the data and different letters indicate significant difference among groups.

Thyroxin (T_4_) is an important constituent of FBS used in cell culture experiments. In this study, the effect of T_4_ on the expression of genes involved in myogenesis was investigated. *TTR* and *Cav1.1* expression were enhanced by T_4_ treatment, while this effect was small in other genes (Fig. 6A). In wild type cells (TTR_wd_), wider myotube formation was observed after T_4_ treatment. However, there was no apparent change in the morphology of TTR_kd_ cells with or without T_4_ treatment (Fig. 6B). Changes in the T_4_ concentration of cells were measured by ELISA (Fig. 6C). A gradual increase in T_4_ concentration was observed in TTR_kd_ cells. In contrast, the concentration of T_4_ in TTR_wd_ cells increased up to day 4 and then decreased at day 6. Interestingly, the intracellular T_4_ concentration of TTR_wd_ cells was significantly higher than that of TTR_kd_ cells at day 4, when C2C12 cells showed peak myotube formation. The increase in intracellular T_4_ concentration can be assumed to be due to the presence of T_4_ in cell culture media and its transport across the membrane.

## Discussion

### TTR-interaction during Myoblast Differentiation

*TTR*, which is known as a carrier protein for thyroxin and retinol binding protein, has been shown to play a critical role in homeostasis of the nervous system [34]. We previously identified *TTR* as one of the genes highly up-regulated in different depots of bovine muscle tissue during myogenesis [30]. This was a novel finding, as the protein had previously been reported as a systematic precursor to deposition and amyloid fiber formation. Immunohistochemical analysis was used to detect the presence of *TTR* protein in skeletal muscles of the forelimb, hind limb and trunk, as well as in the liver (control). The presence was further confirmed by mRNA expression of *TTR* in different muscle depots (Fig. 1). This study describes the role of *TTR* as a key factor in myoblast differentiation. In this study, shRNA that silenced *TTR*, was used to investigate (i) inhibition of myotube formation based on *MYOG* and *MYL2* mRNA expression (ii) changes in mRNA expression of the calcium channel related genes, *STIM1, Orai1, Cav1.1* and *Cav3.1,* and (iii) time dependent occurrence of voltage-gated calcium currents during myogenesis. Our results are in accordance with Mock et al [35], in which they demonstrate the decrease in muscle mass of a *TTR* null mouse.

To investigate the function of *TTR* in myogenesis, C2C12 cells were transfected with shRNA against *TTR* and observed for changes in cell morphology and gene expression. Microscopic observations revealed a reduction in myotube formation in *TTR* silenced cells when compared with wild type (wd) cells. These findings were supported by decrease in *TTR* mRNA and protein expression observed by real time RT-PCR and immunostaining, respectively. Muscle differentiation is accompanied by specific alterations in the pattern of muscle specific gene expressions [36], particularly those of two groups of transcription factors, the *MyoD* family (including *Myf5*, *MyoD*, *MYOG*) and the *MEF2* family. To confirm this, the mRNA expression of these genes was compared in TTR_kd_ and TTR_wd_ cells. *Myf5* was unaltered, whereas variation in the expression of *MYOD*, *MYOG* and *MYL2* was observed. *Myf5* and *MyoD* are known to exist in the early stages of myoblast differentiation [37], while *MYOG* is expressed throughout myotube formation and *MYL2* is expressed at a later stage. As shown in Fig. 2 and 3, *TTR* silencing affects the expression of *MYOG*, which can be assumed to be a result of the early interference of *TTR* during myogenesis. The effect of *TTR* on *MYL2* also confirms its interference during myogenesis. The time course study revealed that the expression of *MYOG* and *MYL2* was highest on day 4, after the culture medium was replaced with differentiation medium. To confirm the role of *TTR* in myogenesis via involvement of the calcium channel(s), the mRNA expression of *STIM1*, *Orai1*, and VGCCs was studied in detail. Darbelly et al. [38] reported that *STIM1* and *Orai1*-dependent store operated calcium entry (SOCE) plays a crucial role in the regulation of myogenesis in human myoblasts. Specifically, they found a correlation between the amplitude of SOCE and *MYOG*/*MEF2* expression and reported that SOCE was the limiting factor in the signaling cascade that controls the fate of myoblasts. However, SOCE amplitude is regulated by *STIM1* and *Orai1*. In the time course study, *Orai1, Cav1.1,* and *Cav3.1* were found to be up-regulated, with their expression peaking at day 4. However, the expression of these genes was down-regulated in TTR_kd_ cells when compared to TTR_wd_ SOCE and VGCC calcium influx functions in reciprocal mechanism pathways. Nevertheless, excitable cells have been found to express SOCE proteins, but contribute little to Ca^2+^ influx [39], [40], whereas non-excitable cells express VGCC proteins but lack voltage-gated Ca^2+^ currents [41], [42]. The decrease in mRNA expression of SOCE genes, namely *STIM1* and *Orai1*, and the mRNA expression of VGCC genes, namely *Cav1.1* and *Cav3.1*, draws attention to the crucial role played by *TTR* during myogenesis. These results suggest that *TTR* initiates myogenesis in both excitable and non-excitable cells.

### Effect of *myogenin* Silencing

Most studies conducted to investigate the process of differentiation have focused on the expression of *MYOG*, a marker gene involved in myogenesis. In the present study, C2C12 cells were transfected with shRNA against *MYOG* and an expected change in cell morphology and decrease in myotube formation was observed. While MYOG_wd_ cells showed well-established myotubes, no myotubes were observed in the MYOG_kd_ cells. mRNA analysis revealed a decrease in the expression of *MYOG* and *MYL2*; however, *TTR* remained almost unaffected, indicating that *TTR* may remain upstream of *MYOG* during differentiation. Surprisingly, real time RT-PCR analysis revealed a decrease in the mRNA expression of *STIM1*, *Orai1* and *Cav1.1*, while *Cav3.1* showed increased expression. These findings indicate that *MYOG* acts during the initial stage of myotube formation. While studying the ability of mononucleated myocytes to synthesize DNA during myogenesis, Andrés. [43] found that the induction of *MYOG* protein precedes that of p21. DNA synthesis revealed that *MYOG* is expressed in the premitotic state of myocytes and p21, a marker of the postmitotic state of myocytes. The decrease in expression of *STIM1*, *Orai1* and *Cav1.1* involved in myogenesis due to silencing of *MYOG* can be assumed to occur in conjunction with the early expression of *MYOG*. Immunocytochemical analysis revealed that less *MYOG* was present in the nuclei. However, real time RT-PCR analysis revealed that *TTR* was unaffected, while *STIM1* and related genes were greatly influenced by *MYOG* silencing. It is speculated that: i) *TTR* is upstream to *MYOG* and ii) *MYOG* either directly (such as through gene regulation by attachment to the *STIM1* promoter) or indirectly (by some unknown pathway) induces the *STIM1* expression, which subsequently works through *Orai1* and/or iii) the early expression of *MYOG* had a direct effect on VGCCs to influence myotube formation. The expression of *TTR* and its silencing effect on *MYOG*, *STIM1, Orai1* and VGCC related genes suggests that *TTR* is the key regulator during myogenesis and activate the *MYOG* transcription factor through some unknown mechanism. However, a detailed investigation is needed to determine exactly how *TTR* affects *MYOG* and how *MYOG* silencing affects the mRNA expression of *STIM1*, *Orai1* and *Cav1.1*.

### *TTR* Influences VGCCs

Calcium signaling plays an important role in many cellular processes including cell growth and differentiation, and Ca^2+^entry into skeletal muscle fiber has been known to contribute to calcium signaling. It has been suggested that T-type calcium current (I_Ca,T_) in developing muscles is involved in pacemaker-like activity while L-type calcium current (I_Ca,L_) could serve as an early contraction triggering mechanism and/or initially to fill and maintain the intracellular calcium stores [44]. In this study, we revealed a role of VGCC during C2C12 cell differentiation. Using TTR_kd_, we found that *TTR* regulates the amplitude of VGCC during C2C12 cell differentiation. The effect of TTR_kd_ was observed in all cells tested, regardless of the time spent in cultures. It has been reported that the *TTR*-induced increase in Ca^2+^ resulted exclusively from an influx of extracellular Ca^2+^ across the plasma membrane, mostly via L- and N-type VGCCs [45]. Also Ca^2+^ concentrations in myoblast cells upon treatment with the nifedipine, an L-type Ca^2+^ blocker has been found to inhibit the myogenic differentiation [46]. Mibefradil, a T- type Ca^2+^ blocker had been found responsible for 57% inhibition of cell fusion. However, mibefradil blocks I_K(DR)_, I_(h-eag),_ and I_K(IR)_ in addition to I_ca(T)._ Since it is known that blocking 50% of I_K(IR)_ reduces cell fusion by only 25%, it was speculated that the 32% inhibition in cell fusion by mibefradil was either an isolated or combined effect of I_K(DR)_, I_(h-eag),_ or I_ca(T)_ [47]. In the present study, I_ca(T)_ was detected before the concentration of the serum was reduced. However, the detection rate increased and reached 100% in fused cells at day 3. The I_Ca(L)_ current was not detected in the early stage, but there was a drastic increase in cells with I_Ca(L)_ on day 2, after which the myocytes started to fuse. A gradual increase in the detection rate was found thereafter. Chronic application of 100 µM of nifedipine, a concentration that fully inhibits Ca_V_1.1, sharply affected the C2C12 myoblast fusion. RT-PCR experiments revealed that the expression of *Cav1.1* and *MYOG* decreased, while that of *TTR* remained unaffected. Repeating patch clamping experiments with cells transfected with vector (TTR_wd_) and shRNA against *TTR* (TTR_kd_) revealed a decrease in the detection rate of both T- and L- types in TTR_kd_, indicating that *TTR* plays a clear role in initiation of and during the cell differentiation process. It has been reported that SOCE in skeletal muscle requires both *STIM1* and *Orai1*, and SOCE and VGCC represent two distinct and independent molecular entities [39]. However, in this study, the silencing of *TTR* and *MYOG* was found to lead to a decrease in both SOCE proteins (*STIM1* and *Orai1*) and *Cav1.1*. These results demonstrate the crucial role of *TTR* during myogenesis in both excitable (voltage-gated calcium entry) and non-excitable (store-operated calcium channel) cells. Accordingly, a detailed study is required to understand this relationship of *TTR* with excitable and non-excitable cells.

### Effect of Thyroid Hormones

Thyroid hormone (T_4_) plays an important role in cellular development, differentiation and metabolism [48], [49], [50]. Specifically, T_4_ regulates gene expression mediated through thyroid hormone receptors in the nucleus of target cells. *TTR* is one of the three primary T_4_ transport proteins found in serum and has a high binding affinity for T_4_ [51]. *TTR* can both inhibit and enhance T_4_ transport across the membrane. To identify the effects of T_4_ in *TTR* expression and myogenesis, the cells were cultured in differentiation medium supplemented with T_4_ hormone. Based on the mRNA expression of different genes involved in myogenesis, *TTR* was highly expressed and showed a four fold difference in response to treatment with 50 ng/mL T_4_. However, other genes related to myogenesis including *STIM1, Orai1* and *Cav3.1* showed almost no change in response to T_4_ treatment. When cells transfected with shRNA against *TTR* (TTR_kd_) and vector (TTR_wd_) were treated with T_4_, no enhancement of myotube formation in TTR_kd_ cells was observed, while TTR_wd_ cells treated with T_4_ showed a slight increase in myotube size. Moreover, T_4_ concentration analysis showed that the T_4_ in cells was higher on day 4, at which the expression of most genes involved in myogenesis peaked. It could be attributed to the fact that the thyroid hormone is considered as positive regulator of muscle development [52]. This result indicates a direct or indirect role of *TTR* in uptaking T_4_ during myogenesis.

In summary, our work demonstrates a robust inhibition of myoblast differentiation in response to *TTR* silencing in C2C12 cells. The findings presented herein indicate that *TTR* initiates cell differentiation either by influencing the early stage expression of the transcription factor *MYOG* or by directly influencing the SOCE proteins *STIM1 and Orai1* as well as the VGCC proteins *Cav1.1* and *Cav3.1*. The effects of *TTR* on two distinct calcium influx pathways indicate that *TTR* is an essential entity that initiates the process of myogenesis, regardless of whether the cells are excitable or non-excitable in terms of calcium influx.

## Supporting Information

Figure S1 ***MyoD*** **expression in** ***TTR*** **knock-down cells.** A) Decreased cell alignment was seen in TTR knock-down cells (TTR_kd_) as compared to *TTR* wild type cells (TTR_wd_) on day two as seen under phage contrast microscope. B) TTR_kd_ showed reduced mRNA expression of both *TTR* and *MyoD* as compared to *TTR*_wd_ by real-time PCR on day 2 in C2C12 during myogenesis. p value indicates the statistical significance of data and different letters indicate significant difference among groups.
(TIF)

Table S1 **shRNA sequence information.** The table shows a list of shRNA sequence information.
(DOC)

Table S2 **Primer information.** The table shows a list of primer indicating species, gene names, product size, Tm (temperature), and primer sequences (F: forward, R: reverse).
(DOC)

Table S3 **Antibody information.** The table shows a list of antibody used and its clone names.
(DOC)
