# Supplementary figures and images for: Transthyretin Is a Key Regulator of Myoblast Differentiation
Source: PLoS One. 2013 May 22;8(5):e63627. doi: 10.1371/journal.pone.0063627 (PMC3661549; doi:10.1371/journal.pone.0063627)

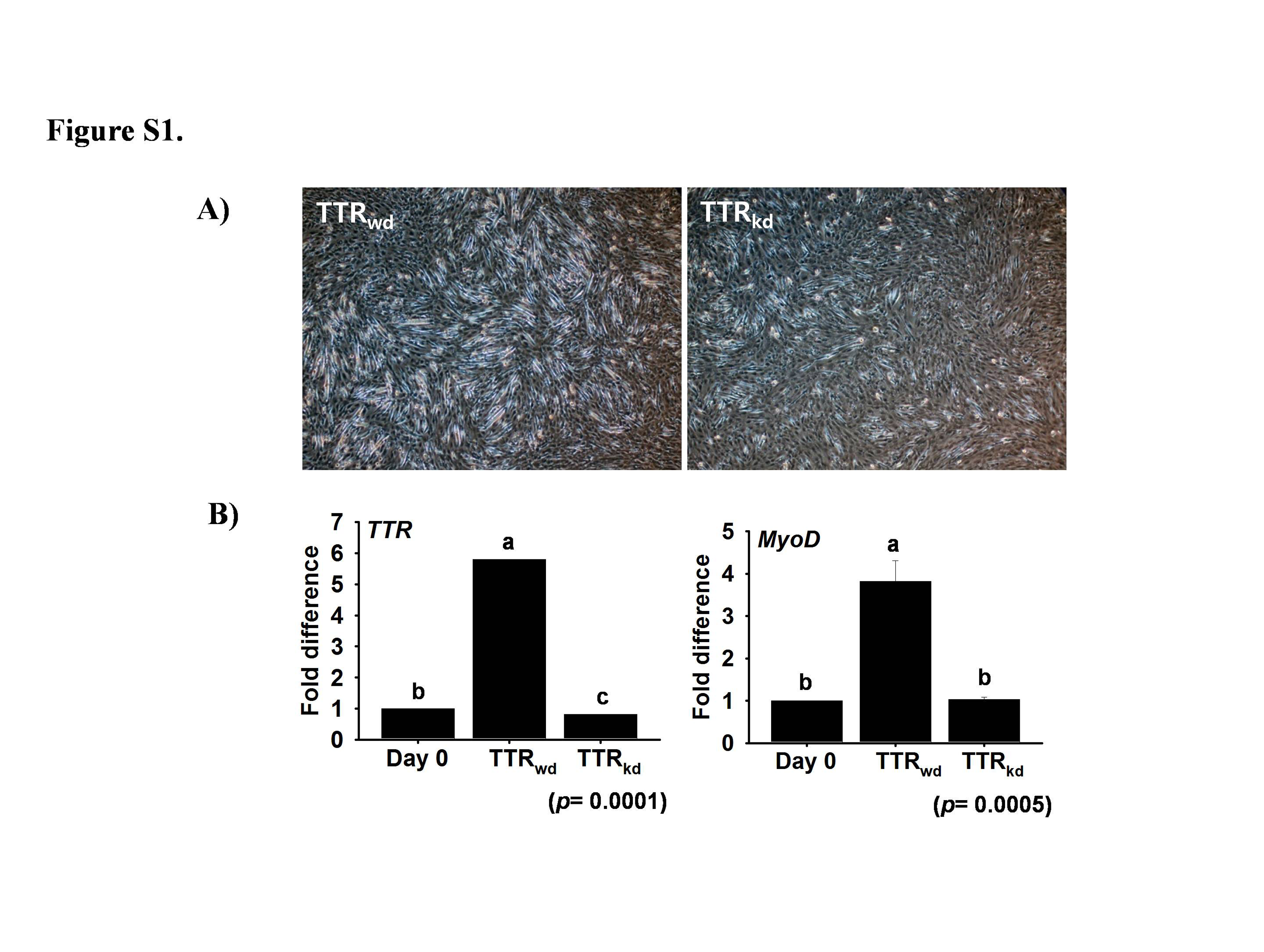

Supplement: Figure S1 — MyoD expression in TTR knock-down cells. A) Decreased cell alignment was seen in TTR knock-down cells (TTRkd) as compared to TTR wild type cells (TTRwd) on day two as seen under phage contrast microscope. B) TTRkd showed reduced mRNA expression of both TTR and MyoD as compared to TTR wd by real-time PCR on day 2 in C2C12 during myogenesis. p value indicates the statistical significance of data and different letters indicate significant difference among groups. (TIF) [file pone.0063627.s001.tif]
