# Supplementary material for: Transthyretin Is a Key Regulator of Myoblast Differentiation
Source: PLoS One. 2013 May 22;8(5):e63627. doi: 10.1371/journal.pone.0063627 (PMC3661549; doi:10.1371/journal.pone.0063627)
Supplement: Table S1 — shRNA sequence information. The table shows a list of shRNA sequence information. (DOC) [file pone.0063627.s002.doc]

**Table S1.**

| **shRNA** | **Sequence** | |
| --- | --- | --- |
| TTRshRNA | Prealbumin shRNA Plasmid (m) is a pool of 3 different shRNA plasmids | |
| (SC-39716-SH) | sc-39716-SHA | GATCCCTGTAGACGTGGCTGTAAATTCAAGAGATTTACAGCCACGTCTACAGTTTTT |
|  | sc-39716-SHB | GATCCGAAGATGCCGTGAAGCATTTTCAAGAGAAATGCTTCACGGCATCTTCTTTTT |
|  | sc-39716-SHC | GATCCCACCTGCTATTTCATTCAATTCAAGAGATTGAATGAAATAGCAGGTGTTTTT |
| MYOGshRNA | Myogenin shRNA Plasmid (m) is a pool of 3 different shRNA plasmids | |
| (SC-35992-SH) | sc-35992-SHA | GATCCGCATGCAAGGTGTGTAAGATTCAAGAGATCTTACACACCTTGCATGCTTTTT |
|  | sc-35992-SHB | GATCCCTGTCCTGATGTCCAGAAATTCAAGAGATTTCTGGACATCAGGACAGTTTTT |
|  | sc-35992-SHC | GATCCCCCATTCACATAAGGCTAATTCAAGAGATTAGCCTTATGTGAATGGGTTTTT |
