# Supplementary material for: Transthyretin Is a Key Regulator of Myoblast Differentiation
Source: PLoS One. 2013 May 22;8(5):e63627. doi: 10.1371/journal.pone.0063627 (PMC3661549; doi:10.1371/journal.pone.0063627)
Supplement: Table S2 — Primer information. The table shows a list of primer indicating species, gene names, product size, Tm (temperature), and primer sequences (F: forward, R: reverse). (DOC) [file pone.0063627.s003.doc]

**Table S2.**

| **Species** | **Gene** | **Product size (bp)** | **Tm (℃)** | **Sequence (F)** | **Sequence (R)** |
| --- | --- | --- | --- | --- | --- |
| Mouse | GAPDH | 155 | 55 | 5'-tgctggtgctgagtatgtcg-3' | 5'-caagcagttggtggtacagg-3' |
|  | TTR | 165 | 59 | 5'-tggacaccaaatcgtactgg-3' | 5'-aattctgggggttgctgac-3' |
|  | Myogenin | 185 | 59 | 5'-tccagtacattgagcgccta-3' | 5'-caaatgatctcctgggttgg-3' |
|  | MyoD | 213 | 59 | 5'-aggagcacgcacacttctct-3' | 5'-tctcgaaggcctcattcact-3' |
|  | MYL2 | 177 | 59 | 5'-aaagaggctccaggtccaat-3' | 5'-cctctctgcttgtgtggtca-3' |
|  | Cav3.1 | 184 | 59 | 5'-tggcaacattgtggtcatct-3' | 5'-gcccaggttgtcaaagttgt-3' |
|  | Cav1.1 | 225 | 59 | 5'-gaccctcttcaccatcgaaa-3' | 5'-ttgctcagcgatgtccagta-3' |
|  | Stim1 | 244 | 59 | 5'-aaccaacaccaccatgacag-3' | 5'-cccttccagatccttcatca-3' |
|  | Orai1 | 182 | 59 | 5'-atggtagcgatggtggaagt-3' | 5'-ttgaccgagttgaggttgtg-3' |
|  | Doi2 | 152 | 59 | 5'-gatgctcccaattccagtgt-3' | 5'-caggtggctgaaccaaagtt-3' |
