# Supplementary material for: Transthyretin Is a Key Regulator of Myoblast Differentiation
Source: PLoS One. 2013 May 22;8(5):e63627. doi: 10.1371/journal.pone.0063627 (PMC3661549; doi:10.1371/journal.pone.0063627)
Supplement: Table S3 — Antibody information. The table shows a list of antibody used and its clone names. (DOC) [file pone.0063627.s004.doc]

**Table S3.**

| **Antibody** | **Type** | **Species** | **Company** | **Cat no.** | **Clone name** |
| --- | --- | --- | --- | --- | --- |
| TTR | Polyclonal | Rabbit | Santacruz | sc-13098 | FL-147 |
| MYOG | Monoclonal | Mouse | Santacruz | sc-12732 | F5D |
| Cav1.1 | Monoclonal | Mouse | Santacruz | sc-21782 | IIF71VH3 |
| Cav3.1 | Polyclonal | Rabbit | Santacruz | sc-25690 | H-300 |
